# Monomorphic Epitheliotropic Intestinal T-Cell Lymphoma: A Case of Extranodal Non-Hodgkin Lymphoma in the Jejunum with Invasion of the Abdominal Wall and Transverse Colon

**DOI:** 10.70352/scrj.cr.26-0280

**Published:** 2026-07-10

**Authors:** Hikaru Ohtani, Tomohide Hori, Satoru Umegae, Takao Iwanaga, Shigeki Nakayama, Ryutaro Nishikawa, Takahiro Shimoyama, Sakurako Suzuki, Toshio Iida, Kanako Saito, Takayuki Yamamoto

**Affiliations:** 1 Department of Surgery, Yokkaichi Hadu Medical Center, Yokkaichi, Mie, Japan; 2 Iida Clinic, Yokkaichi, Mie, Japan; 3 Department of Medical Oncology, Mie University Hospital, Tsu, Mie, Japan

**Keywords:** monomorphic epitheliotropic intestinal T-cell lymphoma, extranodal lymphoma, non-Hodgkin lymphoma, small intestine

## Abstract

**INTRODUCTION:**

Monomorphic epitheliotropic intestinal T-cell lymphoma (MEITL) is a rare extranodal lymphoma, accounting for <1% of non-Hodgkin lymphomas, which most commonly affects Asians. MEITL mainly occurs in the small intestine and is characterized by an aggressive clinical course with a poor prognosis. MEITL shows intractable symptoms and frequently causes perforation and bleeding of the gastrointestinal tract. Bowel perforation and/or bleeding often require emergency surgery.

**CASE PRESENTATION:**

A 77-year-old woman developed left-sided pain nearly at the umbilicus. CT revealed jejunal wall thickening measuring 60 mm. The lesion was hypervascular and showed gradual enhancement. The irregularly shaped tumor invaded the abdominal wall and transverse colon. Endoscopic findings showed a large ulcerative mass accompanied by severe necrosis. Radiographic findings of a small bowel series revealed a long, narrowed segment. Partial resection of the jejunum and planned dissection of the regional lymph nodes were performed, and partial resections of the abdominal wall and transverse colon were performed simultaneously. Histopathologically, diffuse and monotonous proliferation of medium-sized atypical lymphocytes was observed, and invasion into the abdominal wall and transverse colon was confirmed. A definitive diagnosis of MEITL was made based on histopathological assessment and immunohistochemistry. PET/CT revealed multiple areas of increased uptake in the small intestine. Serum levels of soluble interleukin-2 receptor were progressively elevated over time. The patient refused further aggressive treatment for MEITL. She died because of disease progression 142 days after symptom onset.

**CONCLUSIONS:**

Clinicians should be aware that Asian patients who have small intestinal lesions accompanied by refractory symptoms, including bowel perforation and/or bleeding, may have MEITL. Surgeons should work closely with clinicians, and emergency surgery should be considered for suspected bowel perforation and/or bleeding in MEITL patients.

## INTRODUCTION

Histopathologically, the most common subtype of gastrointestinal and colorectal malignant lymphoma is diffuse large B-cell lymphoma.^1)^ Monomorphic epitheliotropic intestinal T-cell lymphoma (MEITL) is a very rare extranodal lymphoma,^1,2)^ accounting for <1% of non-Hodgkin lymphomas.^3)^ MEITL mainly occurs in the small intestine,^2)^ though gastric and colorectal MEITLs have been reported.^1,3)^ MEITL is most commonly diagnosed in Asian populations.^2)^ MEITL is characterized by an aggressive clinical course with a poor prognosis,^1)^ and the median overall survival of MEITL has been reported to be 7–14 months.^2,4–6)^ MEITL shows intractable symptoms, including weight loss and chronic diarrhea.^2)^ Furthermore, MEITL of the digestive tract frequently causes perforation and hemorrhage,^2,7)^ and even an effective chemotherapy regimen may result in bowel perforation and/or bleeding during and after treatment.^2)^ MEITL patients with peritonitis or shock due to bowel perforation or bleeding may require emergency treatment.^3,8)^ Here, we present a case of jejunal MEITL with invasion into the abdominal wall and transverse colon.

## CASE PRESENTATION

A 77-year-old woman developed left-sided pain nearly at the umbilicus, which worsened over 5 days. Her primary physician referred her to our hospital. Blood tests showed elevated C-reactive protein (2.30 mg/dL; normal range, ≤0.30 mg/dL), though other laboratory parameters, including white blood cell count, were within the normal range. The serum level of soluble interleukin-2 receptor was slightly elevated (552 U/mL), though levels of carbohydrate antigen 19-9 and carcinoembryonic antigen were within normal ranges. Dynamic contrast-enhanced CT was performed as part of the initial evaluation (**Fig. 1**), and wall thickening was detected in the jejunum, measuring 60 × 60 mm. The lesion was hypervascular and showed gradual enhancement without early wash-in or wash-out. Swelling of the regional lymph nodes was also detected. The irregularly shaped tumor invaded the abdominal wall and transverse colon. Ascites and abscess formation were not detected. Endoscopic examination revealed a large ulcerative mass with a severely necrotic base (**Fig. 2**). A pathological diagnosis could not be made from 6 biopsy specimens because of the severe necrosis. Radiographic findings of a small bowel series revealed a long, narrowed segment (**Fig. 2**), although the luminal passage remained intact.

**Fig. 1:**
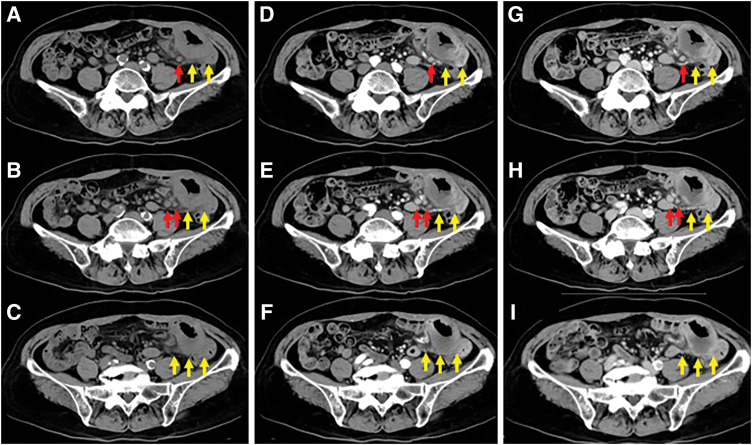
Contrast-enhanced CT findings. Dynamic CT findings in the unenhanced (**A**–**C)**, early phase (**D**–**F**), and delayed phase (**G**–**I**) are shown. Jejunal wall thickening measuring 60 × 60 mm was detected (yellow arrows). The hypervascular tumor was gradually enhanced. Swelling of the regional lymph nodes was also detected (red arrows). The irregularly shaped tumor invaded the abdominal wall and transverse colon.

**Fig. 2:**
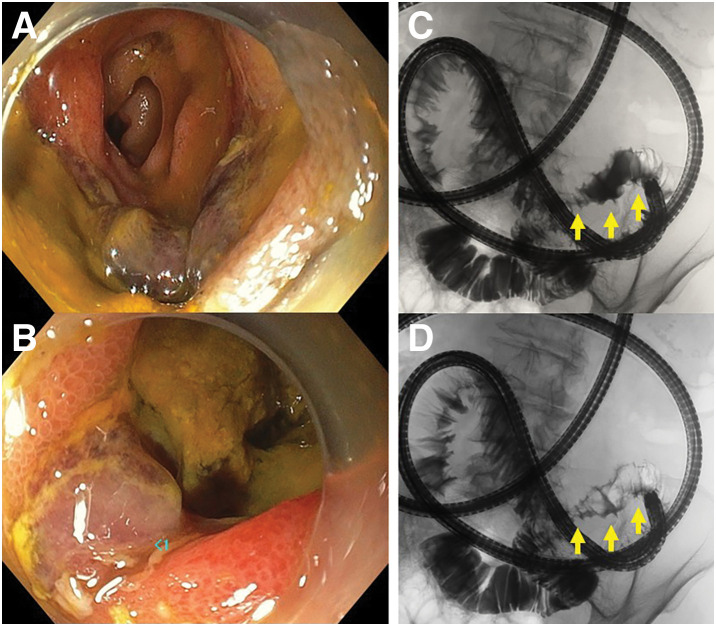
Findings of endoscopic examination and small bowel series. Endoscopic examination (**A** and **B**) revealed a large ulcerative mass with a severely necrotic base. Radiography of a small bowel series (**C** and **D**) detected a long, narrowed segment (yellow arrows).

A preoperative diagnosis of adenocarcinoma or malignant lymphoma was made, and partial resection of the jejunum and planned dissection of the regional lymph nodes were performed. Because the primary tumor invaded the abdominal wall and transverse colon, partial resections of the abdominal wall and transverse colon were performed for optimal *en bloc* resection. The operative time was 69 min, and intraoperative blood loss was 104 mL. The postoperative course was uneventful.

Macroscopically, the jejunal tumor was a large ulcerative mass with a severely necrotic base (**Fig. 3**), and the hard, elastic tumor invaded the abdominal wall and transverse colon (**Fig. 3**).

**Fig. 3:**
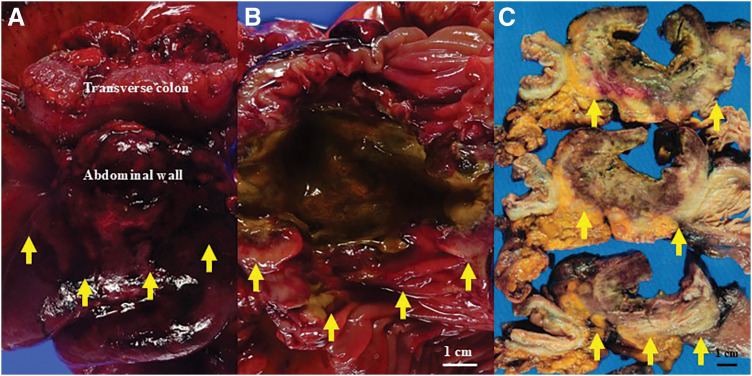
Macroscopic findings of resected specimens. The jejunal tumor (yellow arrows) invaded the abdominal wall and transverse colon (**A**) and was a large ulcerative mass with a severely necrotic base (**B** and **C**).

Histopathologically, diffuse and monotonous proliferation of medium-sized atypical lymphocytes was observed (**Fig. 4**). Direct invasion into the abdominal wall and transverse colon was histopathologically confirmed. No metastases were detected in a total of 22 surgically dissected lymph nodes. In immunohistochemical assays, lymphocytes were positive for CD3, CD7, CD8, CD20 (weakly), CD56, and granzyme B (focally), and were negative for CD4, CD5, CD10, CD30, and TIA-1; Epstein–Barr virus-encoded RNA was also negative by *in situ* hybridization (**Fig. 5**). The Ki-67 labeling index was 80% (**Fig. 5**). A definitive diagnosis of MEITL was made based on histopathological assessment and immunohistochemistry.

**Fig. 4:**
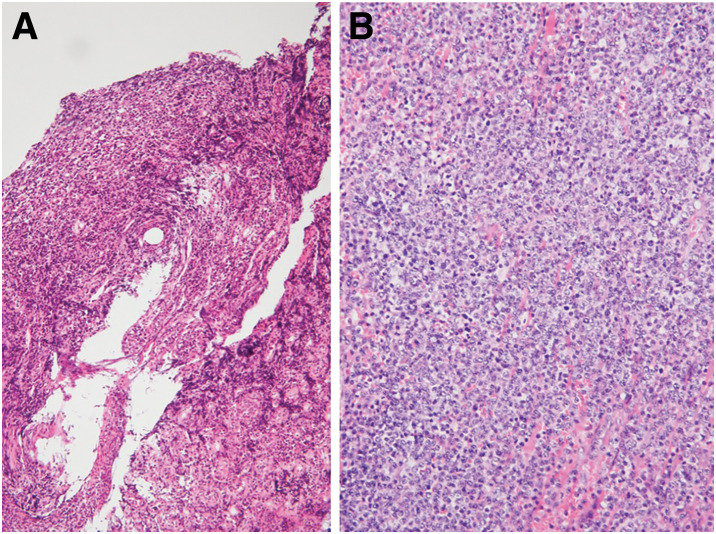
Histopathological assessment. Diffuse and monotonous proliferation of medium-sized atypical lymphocytes was observed. Histopathological findings are shown with hematoxylin and eosin staining at ×40 (**A**) and ×200 (**B**) magnification.

**Fig. 5:**
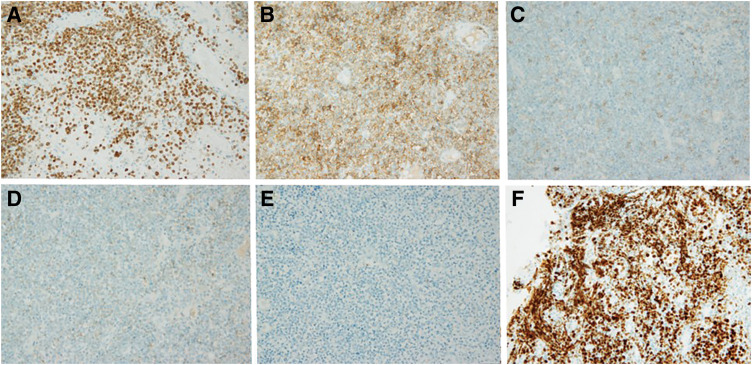
Immunohistochemistry. Atypical lymphocytes were positive for CD3 (**A**), CD8 (**B**), CD20 (**C**), and CD56 (**D**), and were negative for TIA-1 (**E**) by immunohistochemistry. The Ki-67 labeling index was 80% **(F)**.

PET/CT revealed multiple areas of increased uptake in the small intestine (**Fig. 6**). Although the serum levels of lactate dehydrogenase remained within the normal range, serum levels of soluble interleukin-2 receptor increased (552, 713, and 2540 U/mL at 0, 1, and 2 months, respectively; normal range, 157–474 U/mL).

**Fig. 6:**
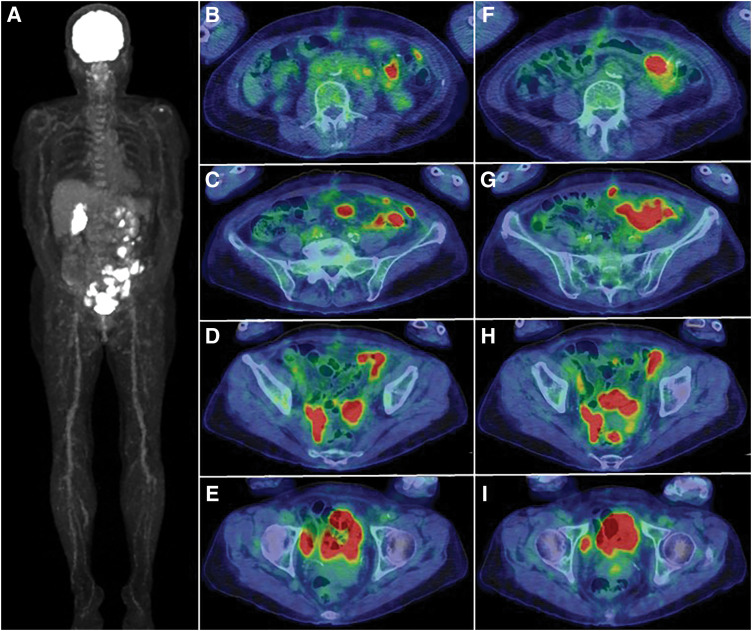
PET/CT. A whole-body PET/CT image is shown (**A**). Multiple areas of increased uptake in the small intestine were detected (**B**–**I**).

The patient and her daughter refused further aggressive treatment for MEITL. She died because of disease progression at 142 days after symptom onset.

## DISCUSSION

MEITL is an extranodal T-cell lymphoma, histopathologically characterized by the proliferation of intraepithelial lymphocytes.^7)^ This subtype of intestinal T-cell lymphoma was formerly known as type II enteropathy-associated T-cell lymphoma (EATL), which is not associated with celiac disease.^9)^ MEITL in the alimentary tract is currently defined by the World Health Organization classification (2017).^9)^ MEITL often has multifocal lesions in the alimentary tract.^2)^

MEITL is characterized by an aggressive clinical course accompanied by refractory symptoms^1,2)^ and has a poor prognosis.^1,2,4–6)^ In MEITL patients treated with chemotherapy and/or surgery, the median progression-free survival and overall survival were 1.0–6.9 and 7.0–14.8 months, respectively.^4,5)^

MEITL shows characteristic findings, including jejunal involvement with diffuse bowel wall thickening and luminal narrowing.^2)^ Endoscopic features in MEITL include edematous mucosal thickening and multifocal shallow ulcerations,^2)^ frequently observed alongside a mosaic mucosal pattern or diffuse erosions.^2)^ Random biopsies of multiple intestinal enteropathy-like lesions with immunohistochemical detection of CD3 and CD8 may help determine tumor spread and confirm prodromal non-neoplastic lesions.^10,11)^ In the current case, a preoperative diagnosis of MEITL could not be made because of severe necrosis in the biopsy specimens (**Fig. 7**). The jejunal tumor in our patient showed the following features on endoscopic examination and small bowel series: (i) a significantly more difference in tissue necrosis compared with the primary adenocarcinoma, (ii) oozing freely upon even subtle contact compared with the primary adenocarcinoma, (iii) an elastic rather than soft neoplasm, and (iv) only light passage obstruction relative to its size. We suggest that these characteristic findings may be informative for making a preoperative diagnosis of MEITL.

**Fig. 7:**
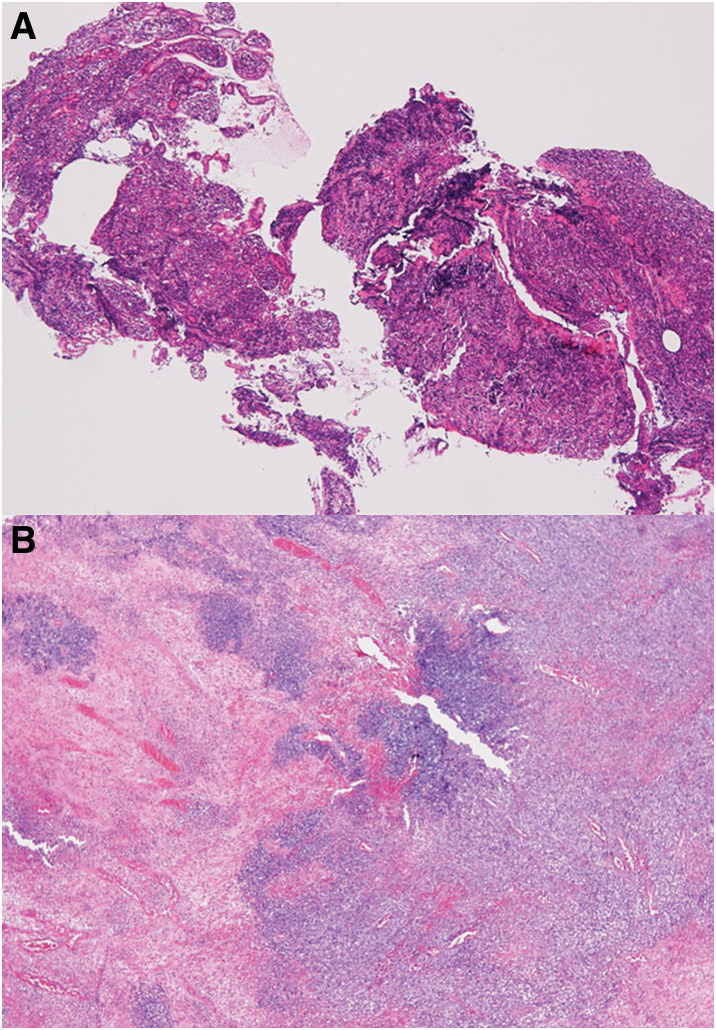
Histopathological assessment of biopsy specimens. No pathological diagnosis could be made based on the 6 biopsy specimens because of severe necrosis (hematoxylin and eosin staining; ×20 (**A**) and ×100 (**B**) magnification).

MEITL most commonly affects Asian populations.^2)^ Our patient was Asian, and she had typical radiographic and endoscopic findings. Clinicians should be aware that Asian patients with small intestinal lesions accompanied by intractable symptoms, including bowel perforation and/or bleeding, may have MEITL.^2)^

From a therapeutic perspective, a prompt definitive diagnosis of MEITL and appropriate treatment are crucial.^12)^ The standard treatment for diffuse large B-cell lymphoma is cyclophosphamide, hydroxydaunorubicin (doxorubicin), oncovin (vincristine), and prednisone (CHOP) chemotherapy comprising cyclophosphamide, doxorubicin, vincristine, and prednisone.^13)^ However, the prognosis of MEITL patients treated with CHOP-like chemotherapy is poor,^2)^ because, unlike B-cell lymphomas, peripheral T-cell lymphomas are resistant to CHOP chemotherapy.^2)^ Prognostic factors include age, performance status, bowel perforation, and anthracycline chemotherapy.^2)^ Multifocal lesions in the small intestine are common in MEITL.^1)^ The clinical role of surgery in patients with MEITL mainly involves emergency treatment for bowel perforation and/or bleeding at initial examination or during chemotherapy.^14,15)^ MEITL generally shows multifocal lesions in the small intestine,^1)^ and might therefore deteriorate rapidly after emergency surgery for bowel perforation and/or bleeding.^14)^ Although elective surgery (i.e., non-emergent surgery) may be a therapeutic option,^3)^ surgical resection is not curative and has no lasting benefit for MEITL patients.^1,2)^ One reported case of multi-refractory MEITL was treated effectively by combined therapy with repeated surgeries and cord blood transplantation.^16)^ Intensive chemotherapy followed by autologous stem cell transplantation may provide a better outcome,^2,8)^ though evidence-based therapeutic decision-making in MEITL patients is extremely challenging.^12)^ Treatment regimens for MEITL have been proposed,^12,17)^ and brentuximab vedotin with chemotherapy is currently available for CD30-positive peripheral T-cell lymphomas.^18,19)^ Surgical resection should never be considered a curative treatment in MEITL,^1)^ and advanced systemic chemotherapy is thus an indispensable therapeutic strategy in these patients.^2)^ Cord blood transplantation or total body irradiation might have been unrealistic choices for our patient.^16,20)^ We therefore proposed a total of 4 cycles of gemcitabine, dexamethasone, and cisplatin, followed by 3 cycles of methotrexate, procarbazine, and vincristine, based on our previous experience,^21,22)^ and because immunohistopathological assessment suggested that this T-cell lymphoma might be resistant to conventional chemotherapy with CHOP.^2)^ Although our patient and her daughter refused advanced chemotherapy, minimal chemotherapy with gemcitabine alone was considered to be safe and feasible, even in this septuagenarian patient.

MEITL shows refractory symptoms (e.g., weight loss, chronic diarrhea)^1,2)^ and tumors generally express cytotoxic molecules and markers^23)^; therefore, MEITL may cause bowel perforation and gastrointestinal bleeding.^2,7)^ Previous reports have described the aggressive clinical behavior of MEITL, and rare cases including direct invasions into the brain and bone marrows have also been documented.^24–26)^ To the best of our knowledge, however, no cases of MEITL accompanied by direct invasion into the abdominal wall and/or colorectal lumen have been reported. Therefore, in our case, we speculated that the jejunal perforation might be subsequently encapsulated by the abdominal wall and transverse colon as a way of repair to prevent a progressive peritonitis. Bowel perforation and/or bleeding often result in generalized peritonitis and shock.^27)^ Gastrointestinal and colorectal surgeons should work closely with internal medicine specialists and support appropriate management of MEITL patients. Emergency surgery should be considered for MEITL patients with suspected bowel perforation or bleeding.

## CONCLUSIONS

Clinicians should be aware that Asian patients who have small intestinal lesions accompanied by refractory symptoms, including bowel perforation and/or bleeding, may have MEITL. Surgeons should work closely with clinicians, and emergency surgery should be considered for suspected bowel perforation and/or bleeding in MEITL patients. We anticipate that this case report will be informative in the field of gastrointestinal and colorectal surgery.

## DECLARATIONS

### Availability of data and materials

All data analyzed during this case report are included in the article.
